# Formulary for the Preparation and Employment of the Salts of Morphine, of Strichnine, Brucine, Cinchonine, Quinine, Emetine, Iodine and Other New Medicines

**Published:** 1828-04

**Authors:** 


### REVIEWS AND BIBLIOGRAPHICAL NOTICES.

---

Art. II.—*Formulary for the preparation and employment of many new Medicines; such as the Nux Vomica, the Salts of Morphine, the Prussic Acid, the Strychnine, the Veratrine, the Alkalis of the Cinchona, the Emetine, the Iodine of Mercury, the Cyadine of Potassium, the Croton Oil, the Salts of Gold, the Salts of Platina, &c. &c. By* F. Majendie, *Member of the French Institute, Titular of the Royal Academy of Medicine and of the Philomatic So-Society, Physician of the Central Office of Admission to the Hospital and Almshouses of Paris, &c. &c. Translated from the fifth edition, revised, and augmented, by* John Baxter, M. D. *Member of the Medical Societies of Philadelphia and New-York. With notes and abbreviations. New-York,* 1827.

It is a curious fact, that the *Confectio Mithridatis* was composed of more articles, than any enlightened physician, of the present day, is accustomed to employ in his general practice! Synthesis, or composition, was the fundamental principle of the ancient pharmacy; while analysis is the object of the modern. By the union of a great number of simples, the pharmacopolists of Greece and Rome, expected to form a medicine of multifarious powers; but, when the seething pot is charged with more than three score and ten decomposable bodies, neither their actions on each other, nor the new compounds, which may spring from the long and complicated series of decompositions, can be foretold. When a variety of simples are thus compelled to make war on one another, many of them must, necessarily, be destroyed; and, in the end, if the farrago be reduced to the state of an electuary, its effects, as a medicine, may be exceedingly limited. Indeed, if we would form a compound, possessing a variety of therapeutick powers, its elements must necessarily be few, and not easily decomposed; and hence, we are indebted to modern chemistry for a knowledge of the means of making prescriptions of a more complex operation, than any which the ancient polypharmacy could boast.

Other, and more important advantages, however, have been conferred on the Materia Medica, by analytical chemistry. By resolving the native vegetable compounds into their immediate elements, it has enabled physicians to make a separate trial of each; and, thereby, to select the portion, in which the active power appears to reside. We have thus been furnished with no inconsiderable number of new and powerful bodies; liberated, like spirits, from the bulky and inert masses in which they were concealed. The medicinal effects of these insulated elements, are, by most physicians, presumed to be identical with those of the drugs which afford them. But this is assuming, what should be proved by experiment. Opium, the Bark, and Laurel water are complicated articles, and the several ingredients in each, no doubt, contribute to their respective effects; although the governing principles may be morphine, quinine, and the hydrocyanic acid. Among all the researches which could be undertaken in the Materia Medica, we know of none, which promise a greater reward, than an inquiry, by means of observation and experiment, into the comparative powers of these newly discovered agents, and the old medicines from which they have been extracted. It is not hazarding much, to say that the qualities of the two classes, are not precisely the same; and we have as little hesitation in predicting, that the former will never entirely supersede the latter. They should be regarded as auxiliaries, rather than substitutes. In some cases the bulky and complicated Galenical will be preferable: in others the concentrated chemical—which will generally present the additional convenience of easier administration, and the advantage of exciting less disturbance of the stomach.

Among those to whom the profession is indebted for information concerning the modus operandi of these powerful bodies, the indefatigable author of the little book now before us, deserves, perhaps, the highest rank. His observations, however, are rather physiological than clinical. In his numerous and diversified inquiries into the economy of living bodies, he has, in various modes, subjected animals to the action of these agents; and, thus, by the same experiments, enlarged the boundaries of both Physiology and the Materia Medica. We have been told that his habit is, to engage in experiments on living animals, with the knife or poisons, every Sunday at 12 o'clock! Researches are made by him on other days, as business may permit; but the Sabbath is consecrated to that object.

Of the relative effects of these deleterious agents on man, and such of the inferior animals as approach to him in their organization, such an experienced physiologist must be well qualified to speak. The following is his testimony:—

"Fifteen years experience in my laboratory, and at the bed side of the sick, enable me to affirm, *that these medicines and poisons act in the same manner upon man as upon the other animals.* My confidence in this respect is such, that I do not hesitate to use on myself the substances which I have found innocent in their effects upon other animals; and I should not advise any one to make an inverse experiment." p. iv. of preface.

In coming to the review of particular articles, we are first presented with the two alkalies—Strychnia and Brucia—obtained from certain plants of the natural order *Strychneæ* of Decandolle. Both these active principles exist in the *Strychnos Nux Vomica*, the *S. Ignatia*, and in the poison called *Upas Tieute;* but Brucia has been discovered, also, in the *Brucea Anti-Dysenterica*, where it is combined with the Gallic acid, and exists distinct from Strychnia. In the plants of the natural order Strychneæ, both the alkalies are united with an acid, to which Pelletier and Cavenou have given the name of Igazuric.

Our earliest professional recollections present the *nux vomica* among the articles of the *shop;* but, as one of deleterious and undefined powers, which was kept closely bottled up, and never employed as a medicine. In 1809 Majendie and Delille, by treating it with highly rectified alcohol, obtained an extract of great activity; which was found to contain both the aforesaid alkalies, with some of the inert principles of the plant. It was with this extract, that they excited those unique effects on the muscular system of animals, which suggested its use in paralytic affections. The subsequent administration of the Strychnia and the Brucia, separated from the other ingredients, and from each other, has proved that it is on them the effects depend; but that the former is far more energetic than the latter. The following is our author's account of the action of the dry alcoholic extract, on animals and on man.

"*Physiological properties.* A grain of this extract, absorbed at any point of the body, or mixed with the food, causes quickly the death of a middling size dog, producing attacks of tetanus, which, by prolongation, impede respiration until complete asphyxia is produced.

When the dose is much stronger, the animal appears to perish by the same action of the substance on the nervous system, as M. Segelas has ascertained. (See my Journal of Experimental Physiology, October, 1822.) Dr. Defermon describes a sort of contraction of the spleen which takes place in animals poisoned by the alcoholic extract of nux vomica. I have myself observed the same phenomenon. When an animal under the action of this substance is touched, he experiences a jerk similar to a strong electric shock. This effect is reproduced by every new contact.

The section of the spinal marrow behind the occiput, and even the complete beheading, does not prevent the effects of this substance from taking place, and even from continuing some time. This character distinguishes the action of the alcoholic extract of strychnos from that of all other substances at present known. The excitement of the spinal marrow is only transmitted to the muscles by the anterior roots of the rachidian nerves: the posterior roots have not the same property. (See Journal de Physiol. Exp. tom. 3.)

After death, no lesion of the tissue, which can point out the cause which produces it, is to be found.

*Action of the alcoholic extract on man in health.* This is identically the same as that which has just been described; and, if the dose is carried high enough, death happens quickly with the same symptoms. The body offers likewise no apparent lesion of the tissue: nothing is observed but traces of asphyxia, which has produced or accompanied death. I ascertained this upon a woman after being poisoned.

*Action upon man diseased.* Upon a man affected with paralysis, the effects are similar to those described; but they have this very remarkable circumstance, that they are particularly exhibited in the paralyzed parts. It is there that occurs the tetanic agitation; it is there that a feeling of prickling announces the action of the medicine; finally, it is there that is developed a local perspiration which is not observed elsewhere. In hemiphlegias, submitted to the action of nux vomica, the contrast between the two halves of the body is striking; while the sound side is quiet the sick sick side experiences extreme agitation; tetanic spasms succeed one another rapidly, and a copious sweat appears. I have seen upon one woman the affected side covered with an anomalous eruption: the opposite side did not offer the slightest trace. The tongue itself presents this difference between the two halves: one experiences often a bitter taste very evident, while the other offers nothing of the kind.

If the dose is carried further, the two sides participate, but unequally, in the tetanic effect, so much so that the patient is sometimes thrown out of bed, so intense are the tetanic paroxysm.

In a feeble dose, the alcoholic extract of nux vomica, like many remedies, has not any action which can be immediately recognised: it is only after a certain number of days that its advantageous or injurious effects can be appreciated.

*Cases in which the alcoholic extract of nux vomica may be employed.* These are all diseases of debility, either local or general; paralysis of all kinds, general or partial. Mr. Edwards cured with nux vomca an amaurosis with palsy of the upper eyelid. I have seen very good effects in marked debility of the organs of generation, incontinence of urine, &c. I have employed also the resin of nux vomica for feeble stomachs, and extreme general debility, with irresistible tendency to sleep. I have recently used it with advantage in several cases of atrophy of the superior and inferior extremities. It is not proper to give to patients the extract of nux vomica, strychnine, or brucine, but at a distant period from that at which the appoplexy took place which occasioned the paralysis; and cure is not obtained in these consecutive palsies, while there is cerebal organic lesion; for, while such lesion exists in any part of the brain having influence on the movements, the palsies which result are incurable, and it would be dangerous to persist in the employment of these remedies." pp. 2—3—4.

The action of Strychnia on the animal economy, is entirely similar to that of the alcoholic extract of nux vomica, only that it is much more energetic. From the twelfth to the eighth of a grain is the proper dose given in a pill; or a tincture of it may be prepared with alcohol.

The action of Brucia is analagous to that of Strychnia, but much less violent. By experiments on animals, it would seem that the difference is as twelve to one; and on this account, it would be well to substitute the former for the latter, as possessing a less dangerous activity.

The articles under consideration, have been, as yet, but seldom employed in the Western country. It is to be regretted that our Druggists are not more forward in the importation of a variety of preparations, which cannot, conveniently, be made by village and country physicians. Most of our practitioners cannot, indeed, even obtain alcohol of sufficient strength, to form a tincture of the nux vomica, in the best manner.

We have used this medicine in a single case only, and then gave it in substance, made into pills. The patient laboured under hemiplegia. He was of a sanguine temperament and full habit—upwards of 50 years old. After great depletion from the blood-vessels and bowels, and subsisting on a reduced diet for several months, his system became, as is common in such cases, exceedingly irritable; and would not bear further reduction. The loss of power in the affected side, remained, however, as entire, as on the day of the attack; and the prospect of advantage from the nux vomica seemed to be considerable. After continuing it for some time, the characteristic spasms came on. They were confined to the side affected; and were much greater in the arm than leg. The muscles were made rigid; and the influence of the will over them was so far restored, that when his fingers were hooked in any heavy body, he was able to draw it towards him, with considerable power. But under the continued administration of the medicine he did not acquire the ability to govern the motions of his limbs; and as they were often affected, especially at night, with painful spasmodic jerkings, it was, after some weeks, discontinued. It may not be improper to add, that although advanced in life, this patient, in two or three years, acquired a tolerable use of his limbs, and is able to walk about the village in which he resides. This appears to be the effect of the mere *lapse of time*, which, in every stage of life—even to old age—accomplishes more in paralytic affections, than in almost any other. We have, repeatedly, seen cases of hemiplegia, extending to the whole side, or confined to the face, that have been removed, so perfectly, as to leave but little decrepitude, after the skill and patience of the profession had been exhausted, and the subjects of them had been abandoned to the *medicine expectante.* When the cerebral plethora, which constitutes the prominent circumstance, in the early stages, is reduced, but little more, perhaps, can be accomplished by art. Such, at least, is our experience.

Opium. This ancient drug has long tried the skill of both chemists and physicians. Being a *succus spissatus*, it, of course, contains all the ingredients of the poppy juice which affords it, and these are not a few. They may be regarded as so many distinct substances, united according to the chemistry of the vegetable kingdom. Several of them are active agents; and, hence the diversity of effects which this medicine presents when administered. Varieties in the constitutions of patients, contribute also to the enigmas which it frequently offers. Thus one man may have a lively susceptibility to one of the elements of Opium, while another may be almost insensible to *its* action, but easily and powerfully excited by a different element. To chemistry, only, can we look for the means of extrication from the labyrinth, in which our predecessors have wandered for ages. The discovery of Derosne, in 1803, was the first decisive step; and, although the progress of experimental inquiry has, ever since, been uninterrupted, the goal is not yet attained. A decided and most useful advancement, however, has obviously been made. In the following paragraph, M. Majendie gives the results of analysis, up to the year 1827:

"It results from the labors of chemists, and particularly from the researches of Messrs. Derosne, Serteurner, Robiquet, and Robinet, that opium is composed, 1st, of a fixed oil; 2d, of a matter resembling caoutchouc; 3d, of a vegeto-animal substance, which has not been sufficiently studied; 4th, of mucilage; 5th, of fecula; 6th, of resin; 7th, of the remains of vegetable fibre; 8th, of narcotine; 9th, of meconic acid; 10th, of the acid meconate of soda; 11th, of the codate of morphine." pp. 18—19.

Of these principles, two give character to the compound. They are Morphia and Narcotin. The former is alkaline; and appears, always, to exist in opium united with an acid into a neutral salt. This, till lately, was considered to be the Meconic; but the researches of Robiquet and Robinet have shown, that soda is united with the Meconic acid; and the Morphia with another acid, denominated Codic—from a Greek word, signifying a poppy head.

The alkaline properties of narcotin are dubious; but it is soluble in some of the acids, especially the acetic.

We intentionally pass over most of what relates to the manipulations by which these principles are obtained. But it may be of practical utility to recollect the tests for opium, which is so often the occasion of death, when taken by design or by accident. The relations of meconic acid with lead, suggested to Dr. Hare the employment of the acetate of that metal, as a test for opium, which *always* contains meconic acid. We refer to his formula, republished in the fourth number of the Western Medical and Physical Journal. The habitudes of morphia afford other tests equally certain, and of more extensive application; as that alkali and its compounds are used, separately, in the practice of physic, when the test which requires the presence of meconic acid, would of course be useless. By nitric acid, morphia is changed to a red colour. With a solution of deuto sulphate of iron, according to Robinet, it strikes a blue colour. In cases of poisoning from opium, it would, obviously, be proper to resort to the whole of these tests; not that an antidote for opium is known, but, because no medical man should give testimony in such a case, till he has availed himself of every practicable means of ascertaining the truth.

The effects of morphia and its compounds are said to differ materially from those of narcotin. The following are the remarks of our author on the—

"*Action of Morphine on man and on other animals.* The pure morphine, being but little soluble, does not easily make apparent what is the narcotic part of opium; however, there now remains no doubt in this respect: direct experiments have often demonstrated it to me. If, for example, a solution of morphine in oil is made use of, the narcotic effects are obtained very decidedly, even in a feeble dose, such as a quarter or half grain; but it is especially when the morphine is combined with acids that it manifests its narcotic effects, probably because salts of morphine are much more soluble than morphine itself.

It is now nearly five years since I employed, for the first time, the acetate, the sulphate, and hydrochlorate of morphine as remedies.—I have ascertained that these salts possess all the advantages which are desired from opium, without its inconveniences." pp. 21—22.

It may be well to transcribe his formulæ, for the administration of the acetate and sulphate of morphia. They are contained in the following extract:

*Of the Employment of the salts of morphine.* I have endeavored, in the officinal preparations of the salts of morphine, to approach as near as possible to the preparations of opium most used; and I at first composed a syrup of morphine according to the following formula:

*Syrup of morphine.*

| | | |
|---|---|---|
| ℞. | Syrup of sugar, perfectly clarified, | 1 pound, |
| | Acetate of Morphine, | 4 grains. |

This forms a syrup which may replace the syrup of diacodium, with much advantage, as that may be said to be arbitrary.

The syrup of morphine is at present generally used in Paris. Its dose is two tea spoonfuls every three hours. Sleep is often procured with a much smaller quantity; for instance, two tea spoonfuls only, in a little warm water, on going to bed.

Syrup of sulphate of morphine is made with the same proportions and taken in the same dose as that of the acetate.

I employ this syrup when the patients are accustomed to the action of the acetate syrup. In general, by varying the alkaline salts of medicines, their action on the animal economy is sustained for a long while, and without increasing the dose.

*Solution of morphine.*

| | | |
|---|---|---|
| ℞. | Acetate of morphine, | 16 grains, |
| | Distilled water, | 1 ounce. |

Add acetic acid 3 or 4 drops, which may replace the liquid laudanum, the drops of Rousseau, the tincture of opium, &c. The dose of these drops is from 6 to 24.

The solution of morphine may be made by using the sulphate of morphine instead of the acetate.

Besides, the acetate and the sulphate of morphine may be used in pills, in powder, in draught, in juleps, in dose of a 1-4 of a grain to 1 grain in twenty-four hours. I have employed them, both in hospital and private practice, even to 4 grains a day, without any inconvenience. pp. 23—24.

While morphia is regarded as the tranquilizing, narcotin is called the irritating principle of opium. Nevertheless it produces stupefaction, though, according to the observations of our author, not true sleep. It is an energetic poison. He found one grain, dissolved in oil, sufficient to kill a dog. The greatest advantage in the employment of morphia instead of opium, results from its containing no narcotin. To secure this advantage, it is only necessary to extract from the drug all the latter principle, which can be done, according to Robiquet, with sulphuric ether; after which, the opium may be digested in diluted alcohol, in the proportions for making the ordinary tincture. The product is denarcotised laudanum; for the preparation of which Dr. Hare has given particular directions in the paper just referred to. Its operation is essentially the same with that of morphia.

Narcotin, or the exciting principle of opium, should be rejected when we seek to tranquilize our patients; but it by no means follows, that it ought to be thrown out of the Materia Medica. Its curative powers are, in reality, not known; as it has seldom been administered by itself; and, as an active ingredient in an old and favourite medicine, it is fairly entitled to a separate exhibition, until its qualities shall be fully ascertained.

Emeta. The roots of the cephealis ipecacuanha, psycotria emetica, some species of viola, and, perhaps, other emetic plants, afford an alkaline element, on which their power to excite vomiting seems to depend. Whether this principle is the same in all, or modified in different plants, has not yet been ascertained. That, extracted from the two vegetables first above named, is what has hitherto been employed in physiological and clinical experiments. We have not ourselves used it. The following is our author's summary of the effects of this substance, as it was at first prepared, in an impure state:

"*Physiological properties of emetine.* On dogs and cats, emetine, in the dose of 1-2 a grain to 2 or 3 grains, produces vomiting, followed sometimes by quite a long sleep. In a stronger dose, 10 grains for instance, the emetine produces on dogs a repeated vomiting, after which the animal is sleepy. But, instead of returning to health, as in the case where emetine is given in a small dose, the animal dies, generally in twenty-four hours. On opening the body, it is found that death is produced by a violent inflammation of the tissue of the lungs and of the mucous membrane of the alimentary canal, extending from the cardiac orifice to the anus. These phenomena bear great analogy to those produced by tartar emetic".—p. 34.

Pure emeta is so active, that two grains will kill a moderate sized dog; and the fraction of a grain excite vomiting in a man. It unites with the tanno-gallic acid into an insoluble and harmless precipitate; and hence its antidote turns out to be the same with that of tartar emetic—any astringent vegetable decoction.

Emeta is said to have a specific action on the pulmonary tissue. It would be more proper to say, that emetic medicines generally, exert a special action on that tissue; for the effect is not confined to emeta. On this subject practical medicine—before the experiments of our author in conjunction with Pelletier—was in advance of physiology; for nauseating medicines were, generally, prescribed in pulmonary affections, and regarded as important remedies. It is in Italy, that this practice, under Tomassini, has been carried to the highest pitch. Tartar emetic is there given in pulmonary inflammations, to a surprising extent; and considered as a counter stimulant that will subdue inflammation, even more successfully than blood-letting. Concerning the efficacy of this intrepid practice, we are by no means so incredulous as some of our brethren; but we believe that its success is owing, quite as much to the specific action of the medicine upon the affected organ, as to its sedative influence over the heart and brain.

Quina and Cinchonia. The extraction from the different kinds of Peruvian bark, of two active febrifuge principles, is decidedly one of the most important achievements of modern chemistry. For a long time, physicians were impressed with the opinion, that the efficacy of those drugs, in the treatment of intermittent fever, depended on the intimate union which they seemed to present, of a bitter and an astringent principle. The progress of chemical science has corrected this impression; and shown, what, indeed, our clinical experiments had already demonstrated, the futility of attempting, by the addition of astringents to our common bitters, to obtain a substitute for the bark.

Quina and cinchonia exist, probably, in all the species of Peruvian bark; but the pale, yellow, and red—those in common use—are the kinds which have been most successfully analyzed. Both alkalies are found in each species; but vary exceedingly in their proportions. In the pale bark the cinchonia greatly predominates; in the yellow there is an equal predominance of quina; while the red affords both, in large quantities; and would seem, therefore, entitled to the preference as a febrifuge. Water is so far from extracting these alkalies, that, according to M. Guerette, the bark which is left after making the common decoction, contains two thirds as much of both, as existed in it originally; a fact that should not be overlooked in the practice of physic.

Both alkalies unite with different acids, and form salts. The union of quina with sulphuric acid, forming a neutral, an alkaline, and an acid sulphate, is that in which we are most interested. Whatever relates to the action of these alkalies and this sulphate, on the animal economy, is of such importance to the profession, that we shall be excused for making the following extended quotation:

"*Action on animals.* The alkalis on which we have been treating were no sooner discovered, than one of the authors of this interesting labor, M. Pelletier, sent me a certain quantity to study its effects on animals. I soon found that these alkalis, as well as the salts just mentioned, were in no ways poisonous, and even that they had no sudden or appreciable action. They could be then, with confidence, tried upon man sick or well.

*Action upon man in health and diseased.* Numerous observations had induced me to consider these two alkalis as possessing the medical qualities of the barks, and consequently as capable of being substituted for them in all cases. Several physicians, among whom I will mention Messrs. Double, Villerme, and Chomel, occupied themselves on the same subject, and their observations conducted them to the same result as mine.

It may be conceived what advantage must result, in the treatment of diseases, to know precisely the dose of an active substance which is employed, and this advantage is no where better marked than in the case in which we are now engaged, since the quantity of alkalis contained in the barks vary prodigiously, according to the nature and quality of the barks which are employed. We are, besides, often very happy to be able to administer this remedy in so small a volume, and under a form not at all obnoxious. It has been seen, in destructive fevers, that patients perish from this alone, not being able to swallow the necessary quantity of barks in powder; others vomit it up after having taken it; some take on excessive purging, so that the powder passes the intestinal canal without producing any effect. In the most favorable cases, it is necessary that the stomach of the patient should analize chemically, so to say, the bark with which it is filled, and from which it must extract the febrifuge principle; but this labor is always difficult and fatiguing, even to the strongest stomachs: it is, then, a real service which chemistry has rendered to medicine, in having found means to make this separation previously.

M. Caventou has informed us of the effects which he constantly produced by the use of the sulphate of quinine during his labors with M. Pelletier on the barks: he had frequent occasion to taste many liquids containing quinine and cinchonine; he experienced a general excitement, resembling that produced by coffee; the analogy to this action was so striking that he had the idea, as well as M. Pelletier, of analyzing coffee, which several physicians recommended in the treatment of fevers. They did not find either cinchonine or quinine in coffee, but a vegetable base easily crystallizable in long, white, silky filaments, amiantaceous, upon which they did not think proper to pursue their researches, because they learnt that one of their colleagues, M. Robiquet, was occupied on the same object, and had even already his labors advanced upon this base, which has been designated since by the name of cafeine, ang. coffeeine. It is to be regretted that M. Robiquet has not published this result.

The employment of the sulphate of quinine is now generally extended, and its efficacy in fevers of an intermittent type is more and more confirmed. Cases of intermittent fever cured by this mean have been published in all the collections of works of academies and in all journals of medicine. Among the different authors who have written on the subject, and who have spread the use of the febrifuge alkalis, we will mention Dr. Elliotson, (Trans. Med. Chirur. vol. 12. part 2, 1824,) physician of St. Thomas's Hospital, London, who published a very interesting memoir, on the employment of quinine and its sulphate. The pure quinine has given him the same results as the sulphate in intermittent fevers. He has also given this remedy with advantage in intermittent neuralgias and in typhus.—The doses in which this physician administers the quinine and the sulphate are much larger than those in which we give it; he affirms, however, that he has met with constant success. He gave the pure quinine in the dose of 5 grains every six hours: he has even given as high as 10 grains with the same intervals, without any accident.

Dr. Francis Barker, dean of the physicians of the hospital for the treatment of fevers at Dublin, in the Transactions of the College of Physicians of Ireland, has reported thirty cases of intermittent fever of different types which have been cured by the use of the sulphate of quinine. The dose which he gave was from 1 to 3 grains, rarely 4, four times a day. 6, 8, and 10 grains have often been sufficient to prevent the return of fever. However, in some cases, the quantity of sulphate of quinine taken by patients has been 24 to 30 grains, and even in one case it was carried to 44 grains. In the same collection is found a memoir of Dr. John O'Brien, in which this physician reports six cases of typhus treated with sulphate of quinine: in six individuals treated by this mode, (3 to 4 grains a day were given,)

two were cured as promptly as when the sulphate is given in intermittent fevers; in three others the success was less rapid, but as complete; the sixth patient died. We may be astonished, perhaps, to see the sulphate of quinine administered for typhus in England, when theantiphlogistic plan appears so generally adopted in France; but we shall cease to be so, when it is known that I have seen, at the hospital of St. Thomas, in London, Dr. Elliotson administer the sulphate of quinine in large doses for erysipelas, and without accident.

Mr. Bally has also treated, at the hospital de la Pitie, a large number of intermittent fevers with the sulphate of quinine, and always with success.

The efficacy of this remedy is not contradicted in the treatment of putrid fevers. I have reported in my Journal (Jour. de Phys. Exp. July, 1822,) the first examples of putrid fevers so cured by the sulphate of quinine. It was my coadjutor, M. Renauldin, who communicated to me the first fact: I had occasion, a short time after, to give the same remedy with success, (same collection, Oct. 1821,) and now there is no longer a doubt of the utility of this alkali, and the precious advantages which induce a preference of the quinine and its salts to all the other preparations of the bark. M. Dupre, (same Journal, April, 1822,) health officer at Cerisiers, Messrs. Ribes (same Jour. Oct. 1822,) and Piedagnel, (same collection, April, 1822,) have published interesting cases of neuralgias cured by sulphate of quinine: and since, the efficacy of this remedy has been still confirmed in a great number of similar cases.

But it is not only in simple and putrid intermittent fevers, and in neuralgias, that the employment of the sulphate of quinine has been advantageous.

Dr. Klokow (Journal der Practishen Heilkunde, June, 1824,) has arrived, by means of the sulphate of quinine, to arrest, in a woman fifty years old, a veryconsiderable hemorroidal discharge, which had endangered the patient's life. He gave 4 grains of the sulphate at a time: after the sec ond dose the discharge was arrested. The mineral acids, ipecacuanha, and opium, had been employed without success.

Dr. Goupil has treated a man twenty-eight years of age, affected with a severe disease of the chest, with hemoptysis of an intermittent type, and cured him by giving him 18 grains of sulphate of quinine in twenty-four hours, after having applied, two days before hand, 15 leeches to the anus, (Nouv. Bibl. Med., July, 1824.)

We will add still farther, that M. L. Martinet (Revue Medicale, March 1824) has published a memoir on the use of the sulphate of quinine in large doses, in cases of intermittent fevers observed in Italy. It will follow, according to this physician, from the facts which he reports, that the sulphate of quinine, administered in the dose of 12 to 18 grains, in Italy, in quotidien and quartan fevers, did not suppress the paroxyms; but that, given from 20 to 24 grains, it stopped these same fevers completely: that it did not produce any disadvantageous effect upon the abdominal viscera, and that the patients were cured. M. Chomel has given the sulphate of quinine in dose of 36 grains for a single dose with success. We are going to show here some of the results obtained by Italian physicians.

Prof. Mathœis has treated with the sulphate of quinine thirty-one patients affected with tertian fevers, single or double: he has obtained a cure of them; but he was obliged, he says, to carry the dose from 15 to 35 grains in two or three days. This physician reports, also, two cases of putrid fevers cured, one by the bark, the other by the sulphate of quinine.

M. de Rossi has treated sixty-four persons, affected with intermittent fevers of different kinds, and of various types, by means of sulphate of quinine: 8 tertian fevers, 29 double tertian, 2 quartan fevers, 27 subcontinued, and 8 putrid were cured; 50 patients had no paroxysms after the first dose, 7 had them very light. The quantity of sulphate given varied from 12 to 72 grains; but, in twenty-four cases, 24 grains were not exceeded.

The results obtained by M. Tonelli also deserve to be quoted.—He has reported 65 cases of intermittent fevers cured, with the ex ception of one patient only, to whom the sulphate was administered when he was despaired of. Here is the detail of cases reported: 4 quotidien fevers, 22 tertian, 31 double tertian, 3 quartan, 2 double quartan, 2 subcontinued, 1 putrid intermittent. 42 patients had no paroxysm after the administration of the first dose. The quantity of sulphate given to each person varied from 12 to 18 grains." pp. 47—48—49—50—51—52.

Of the new medicines, formed from the Bark, we have employed only the sulphate of Quina; but have used this in a variety of cases. Its effects have been, invariably, those of a tranquilizer. The pulse has become slower and softer, nervous irritability has been allayed, and perspiration promoted. The last—when circumstances favourable to diaphoresis have been present—has seemed to be a prominent effect. To say that the sulphate of quina cures intermittent fever, hæmicrania or, indeed, any other disease, by its stimulating properties, is an averment without proof. We are quite convinced, that it may be administered with advantage in many phlogistic states of the system, provided sudorifics be conjoined; while, in conditions of great debility, it is equally beneficial, if united with active stimulants. It seems, indeed, like the sulphate of morphia, (with which it has many analogies) to exert a powerful influence on the sensibility and contractibility of the general system; bringing them under its own influence; and, thus, superseding morbid actions. Hence it may do good, when the powers of the system are either exalted or depressed; but, in the latter case, it generally requires exciting adjuvants. In many instances, where it seems to give strength, it, in reality, but removes the disease which induced weakness. As a febrifuge, it is unequalled; but, as a restorative, the bark, in substance with aromatics, is, perhaps, preferable.

[TO BE CONTINUED.]

